# Oral Tuberculosis: A Rare Manifestation of Disseminated Disease in a Patient with Dermatomyositis on Chronic Corticosteroids

**DOI:** 10.1155/2016/8193178

**Published:** 2016-11-08

**Authors:** Dina Khateeb, Mohleen Kang, Eugenio Capitle, Mirela Feurdean

**Affiliations:** Department of Medicine, Rutgers New Jersey Medical School, Newark, New Jersey, USA

## Abstract

Tuberculosis remains one of the leading causes of death around the world despite advancements in diagnostic testing and medical therapies. It commonly affects the lungs, but isolated extra pulmonary clinical manifestations have been reported. Tuberculosis of the oral cavity is exceedingly rare. We present a case of a patient with dermatomyositis on chronic steroid therapy, who presented with tuberculosis involving the tongue as the only clinical manifestation of disseminated disease. Physicians must be aware of extra pulmonary manifestations of tuberculosis in patients at risk, in order to avoid delays in diagnosis and treatment and to prevent further contagion.

## 1. Introduction

Tuberculosis is the second greatest global cause of death due to a single infectious agent,* Mycobacterium tuberculosis*. The World Health Organization reported an incidence of 9.6 million global cases of tuberculosis in 2014, affecting mostly South-East Asia and Western Pacific nations [1]. In the United States, tuberculosis usually occurs due to reactivated latent disease in foreign-born individuals and recent immigrants [2]. Additionally, physicians should maintain a high index of suspicion for tuberculosis in specific patient populations with increased risk for tuberculosis, such as the HIV infected and the immunosuppressed. Tuberculosis commonly involves the lungs; however, the disease may affect many organs and may manifest initially with extra pulmonary symptoms.

Tuberculosis of the oral cavity is an exceedingly rare manifestation of disease with an incidence of 0.5–1.5% [3]. We report a case of a Haitian born patient with dermatomyositis on chronic steroid therapy, presenting with lingual disease as the only symptom of disseminated tuberculosis. It is important for physicians to be aware of extra pulmonary manifestations of tuberculosis in patients at risk in order to avoid delays in diagnosis and treatment and to limit further contagion.

## 2. Case Presentation

A 59-year-old Haitian female with history of biopsy confirmed dermatomyositis (initially she presented with lower extremity weakness and creatinine kinase of 6547 u/L), on chronic steroid therapy (prednisone 30 mg daily) for six months, presented to the emergency room with a painful tongue lesion for two months and right shoulder pain. The patient described a four-month history of progressive right shoulder pain exacerbated by movement. An X-ray of the shoulder was unremarkable fourth months prior; however, a recent outpatient magnetic resonance image (MRI) of the right shoulder was suspicious for septic arthritis of the right acromioclavicular joint, and this prompted the current admission. In addition, she reported a tongue ulcer which started after biting her tongue and had progressed in size over the course of two months. She had been treated with a full course of oral acyclovir followed by famciclovir, without improvement. Her travel history included a visit to Haiti one year prior. The patient denied constitutional symptoms of fever, chills, night sweats, or weight loss. She had no cough, shortness of breath, hemoptysis, chest pain, or joint or back pain. She denied any gastrointestinal, genitourinary, or neurologic complaints.

**Figure 1:**
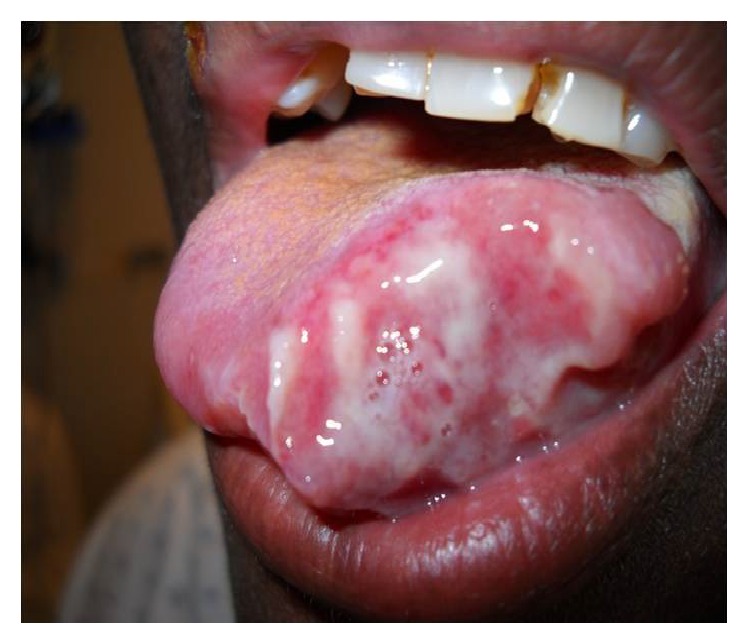
Three by three centimeter ulcerative lesion of the left lateral aspect of the tongue.

On examination, the patient was found to have a temperature of 100.3° Fahrenheit, heart rate of 120 beats per minute, a blood pressure of 135/65 mmHg, a respiratory rate of 18 per minute, and an oxygen saturation of 98% in ambient air. She had a heliotrope rash, shawl sign, and Gottron's papules consistent with dermatomyositis. Head and neck examination revealed soft and mobile left anterior cervical adenopathy with the largest node measuring approximately 1 cm. On the tongue there was a tender, three by three centimeter fungated and ulcerated lesion on the left lateral aspect, with irregular indurated borders and a foul smelling white exudative material (Figure 1). The lungs were clear on auscultation. A musculoskeletal examination demonstrated limited range of motion in the right shoulder due to pain associated with tenderness, warmth, and mild effusion. Serum chemistry was normal. WBC count was 7 × 10^3^/*µ*L with 86% neutrophils and 10% lymphocytes but otherwise the CBC was normal. She was hyperglycemic, with HbA1c of 11.1% (newly diagnosed steroid induced diabetes). She was HIV negative.

In the presence of known dermatomyositis, the nonhealing oral ulcer raised concerns for malignancy. Therefore, a biopsy of the tongue lesion was performed under local anesthesia. Pathology of the tongue biopsy was negative for carcinoma and demonstrated extensive ulceration with granulation tissue and noncaseating granulomas; AFB stain performed on the tissue returned positive for mycobacteria. Periodic acid-Schiff stain was negative for other microorganisms.

**Figure 2:**
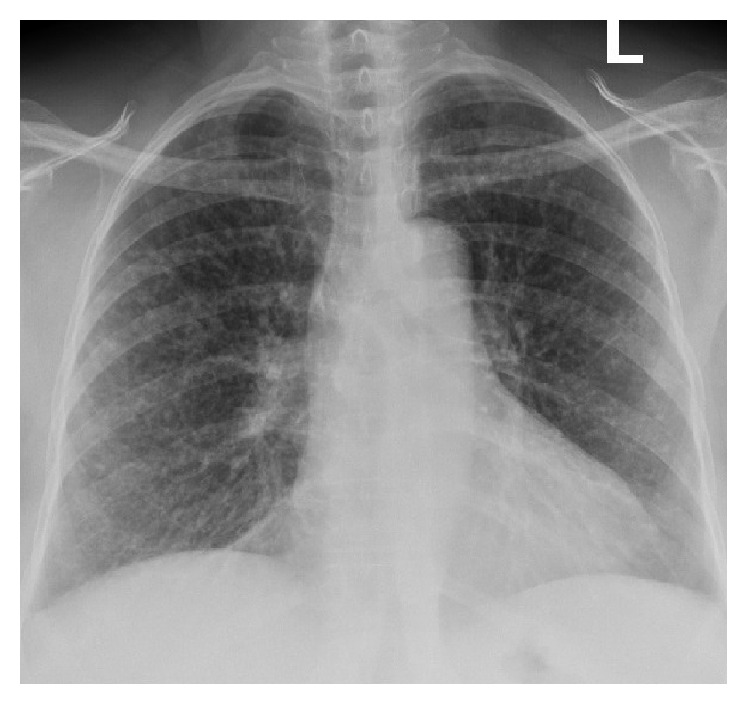
Chest radiograph demonstrating diffuse micronodular densities consistent with a miliary pattern.

Given the positive AFB stain, we reviewed a routine chest radiograph which had been obtained in the emergency room and it revealed diffuse micronodular densities (Figure 2). In contrast, the patient's chest radiograph obtained four months before demonstrated no consolidations or lesions. The patient was placed on airborne precautions and three induced sputum specimens were obtained. Induced sputum specimens returned positive for acid-fast bacilli (AFB), and culture demonstrated* M. tuberculosis*.

**Figure 3:**
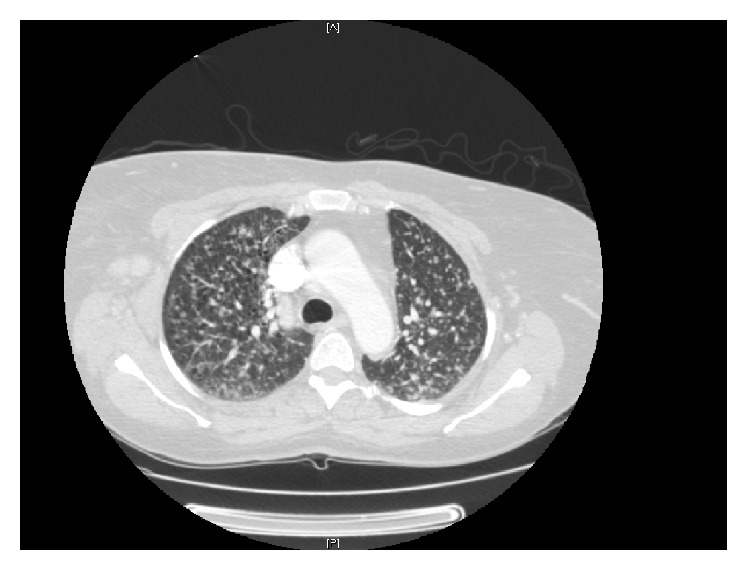
CT Scan of the chest showing miliary pulmonary nodules.

In further evaluation of her pulmonary disease, a computerized tomography (CT) scan of the chest was obtained which showed diffuse miliary nodules and an incidental finding of focal central sclerotic lesions throughout the thoracic vertebral bodies (Figure 3). Therefore CT scans of the cervical, thoracic, and lumbar spine were also obtained and they were consistent with intraosseous tuberculosis. The MRI findings in the right shoulder were presumed to be due to tuberculosis arthritis; repeated joint aspiration was insufficient for microbiological confirmation.

**Figure 4:**
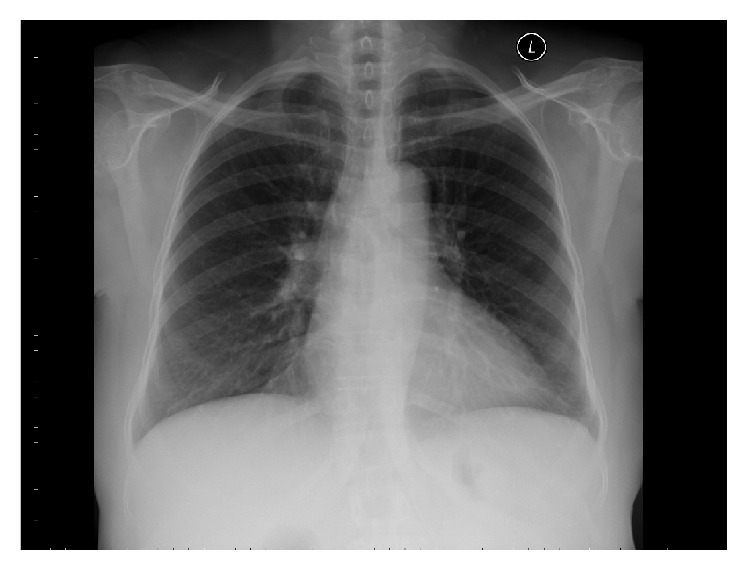
Chest X-ray at 12-month followup with complete resolution of miliary pulmonary nodules.

The patient's prednisone was decreased. She was started empirically on quadruple antituberculous therapy (rifampin, isoniazid, pyrazinamide, and ethambutol with the addition of pyridoxine). Culture sensitivity revealed a pan-susceptible strain of* Mycobacterium tuberculosis*; therefore ethambutol was discontinued. Once the patient completed the initial phase of treatment, she was maintained on isoniazid and rifampin for a total of 12 months. At a five-month followup visit, the tongue lesion was noted to have completely resolved and repeat imaging of her right shoulder demonstrated improvement. A followup chest X-ray at the end of the 12-month treatment showed complete resolution of the previously described miliary nodules in the lung parenchyma (Figure 4).

## 3. Discussion

Tuberculosis is a major infectious cause of global morbidity and mortality that primarily affects the lungs. There are many documented cases of extra pulmonary tuberculosis; however, infection of the oral cavity is exceedingly rare [3, 4]. The oral cavity is an unlikely site for* M. tuberculosis* inoculation due to the inhibitory properties of saliva on mycobacteria [5]. Additionally, the epithelium of the oral mucosa serves as a natural barrier to infection. Therefore, tuberculosis of the oral cavity rarely occurs as primary disease and often arises secondary to infected respiratory secretions or hematogenous dissemination from pulmonary involvement [4–7]. Trauma, as the patient reported in this case, or inflammation of an area in the oral cavity due to smoking may serve as a predisposing factor for either primary or secondary disease [4]. It is thought that our patient suffered hematogenous dissemination from pulmonary involvement, as she had other presumed sites of involvement in the right shoulder joint and vertebrae.

The most common site for oral tuberculosis is the tongue, and the typical presentation is an ulcerative tender lesion, as was demonstrated in our patient. Odynophagia, dysphonia, halitosis, and excessive salivation are other common symptoms of oral tuberculosis [3, 4]. Systemic symptoms of weight loss, anorexia, fever and night sweats may also be present.

Tuberculosis of the oral cavity poses a diagnostic challenge due to its rarity and ability to mimic the appearance of many different conditions such as malignancy, granulomatosis with polyangiitis (formerly Wegener's), actinomycosis, mycotic infections, syphilis, sarcoidosis, Crohn's disease, tongue mycoses, and cat scratch disease [3, 4]. The diagnosis relies on the combination of tissue histology, microbial staining, and culture or polymerase chain reaction. A biopsy is essential to rule out malignancy and other causes of granulomatous lesions, as was the case in our patient. Superficial biopsies may not be sufficient and instances of cancer coexisting with tuberculosis have also been reported [4]. Tuberculin skin testing may yield a false negative result when the tuberculosis is isolated to the oral cavity. Given the hematogenous spread in most cases of oral tuberculosis, further investigation for other sites of infection is also warranted especially pulmonary involvement.

Physicians must acknowledge tuberculosis as part of the differential diagnosis for oral lesions especially in cases where oral lesions fail to respond to therapy. A high index of suspicion is warranted in patients that present with oral lesions and risk factors for tuberculosis. Patients with human immunodeficiency virus (HIV), vitamin D deficiency, silicosis, end stage renal disease, diabetics, smokers, alcoholics, and patients on TNF antagonist therapy or corticosteroids are all at increased risk [8]. It is important to screen rheumatologic patients prior to the start of corticosteroids and disease modifying antirheumatic drugs and biologic agents, as the risk for tuberculosis is doubled in this patient population [9, 10]. Screening with tuberculin skin testing or Interferon Gamma Release Assay (IGRA) and ruling out active disease with a chest radiograph are essential. Patients with latent TB infections should be treated prior to starting any immune suppressive regimen. It is also important to test for HIV since this may have treatment implications [11].

Oral lesions have a favorable response to antituberculosis treatment but lesions may take months to resolve completely. Two months of rifampin, isoniazid, pyrazinamide, and ethambutol (RIPE) therapy followed by four or seven months of isoniazid and rifampin are standard of care [12]. During treatment the patients need to be isolated until sputum AFB smears are negative [3, 4].

This case report highlights the necessity for physicians to remain cognizant of the rare manifestations of tuberculosis especially in immunocompromised patients. Acknowledging the possibility of tuberculosis as part of the differential diagnosis for chronic oral lesions may lead to earlier diagnosis and interventions and prevent further transmission of disease.
